# Suppression PCR-Based Selective Enrichment Sequencing for Pathogen and Antimicrobial Resistance Detection on Cell-Free DNA in Sepsis—A Targeted, Blood Culture-Independent Approach for Rapid Pathogen and Resistance Diagnostics in Septic Patients

**DOI:** 10.3390/ijms25105463

**Published:** 2024-05-17

**Authors:** Mirko Sonntag, Vanessa K. Elgeti, Yevhen Vainshtein, Lucca Jenner, Jan Mueller, Thorsten Brenner, Sebastian O. Decker, Kai Sohn

**Affiliations:** 1 Innovation Field In-Vitro Diagnostics, Fraunhofer Institute for Interfacial Engineering and Biotechnology IGB, 70569 Stuttgart, Germany; mirko.sonntag@igb.fraunhofer.de (M.S.);; 2 Interfaculty Graduate School of Infection Biology and Microbiology (IGIM), Eberhard Karls University Tuebingen, 72076 Tuebingen, Germany; 3 Faculty of Medicine, Greifswald University Medicine, Fleischmannstr. 8, 17475 Greifswald, Germany; 4 Max Perutz Labs, Vienna Biocenter Campus (VBC), Vienna Biocenter 5, 1030 Vienna, Austria; 5 Max Perutz Labs, Department of Structural and Computational Biology, University of Vienna, CIBIV Vienna Biocenter 5, 1030 Vienna, Austria; 6 Vienna Biocenter PhD Program, a Doctoral School of the University of Vienna and the Medical University of Vienna, 1030 Vienna, Austria; 7 Department of Anesthesiology and Intensive Care Medicine, University Hospital Essen, University Duisburg-Essen, Hufelandstr. 55, 45147 Essen, Germany; 8 Department of Anesthesiology, Medical Faculty Heidelberg, Heidelberg University, Im Neuenheimer Feld 420, 69120 Heidelberg, Germany

**Keywords:** sepsis, antimicrobial resistances, cell-free DNA, precision diagnostics, next-generation sequencing, suppression PCR, real-time diagnostics, Nanopore sequencing

## Abstract

Sepsis is a life-threatening syndrome triggered by infection and accompanied by high mortality, with antimicrobial resistances (AMRs) further escalating clinical challenges. The rapid and reliable detection of causative pathogens and AMRs are key factors for fast and appropriate treatment, in order to improve outcomes in septic patients. However, current sepsis diagnostics based on blood culture is limited by low sensitivity and specificity while current molecular approaches fail to enter clinical routine. Therefore, we developed a suppression PCR-based selective enrichment sequencing approach (SUPSETS), providing a molecular method combining multiplex suppression PCR with Nanopore sequencing to identify most common sepsis-causative pathogens and AMRs using plasma cell-free DNA. Applying only 1 mL of plasma, we targeted eight pathogens across three kingdoms and ten AMRs in a proof-of-concept study. SUPSETS was successfully tested in an experimental research study on the first ten clinical samples and revealed comparable results to clinical metagenomics while clearly outperforming blood culture. Several clinically relevant AMRs could be additionally detected. Furthermore, SUPSETS provided first pathogen and AMR-specific sequencing reads within minutes of starting sequencing, thereby potentially decreasing time-to-results to 11–13 h and suggesting diagnostic potential in sepsis.

## 1. Introduction

Sepsis, defined as a “life-threatening organ dysfunction caused by a dysregulated host response to infection”, is a major global health threat, with an estimated 48.9 million cases and 11 million deaths per year worldwide [1,2]. Risk factors including diabetes, obesity, chronic diseases, genetic factors, cancer, or age increase probabilities for adverse outcomes [3,4,5]. Besides an individual’s health implications, sepsis generates significant costs for clinical intervention as well as for long-term follow-up care [6,7]. The numbers of bloodstream infections are rising [8]. On top of this, the increasing incidence of antimicrobial resistances (AMRs) leads to additional threats for septic patients, which are not only limited to septic patients. AMRs are claimed to be a silent pandemic with major impacts, e.g., on patients, treatment options, the environment, and animals, underlining the necessity of the One Health approach to face AMRs [9,10,11]. A meta study from the Antimicrobial Resistance Collaborators revealed that 4.95 million deaths were associated with bacterial AMRs in 2019 [12]. O’Neill and colleagues estimate that by 2050, at least 10 million deaths worldwide will be attributable to AMRs, emphasizing the need for bloodstream infection and AMR diagnostics [13].

Early and appropriate treatment improves the outcomes of septic patients, and therefore identifying causative pathogens at the earliest possible time-point is crucial [14]. Currently, culture-based analysis (e.g., blood culture) represents the gold standard to assess pathogenic burden: blood samples are cultivated in culture bottles for several days until a positive result is obtained [15,16]. However, blood culture has several limitations (being contamination-prone, antibiotic pre-treatment impacts, volume, time-to-diagnosis, and slow growing or uncultivatable specimens) including low sensitivity and specificity [17,18]. Molecular-based methods like standard PCR or qPCR on isolated genomic DNA still fail to prove an impact on clinical utility due to ambiguous results [19,20].

Since it was first discovered in 1948 by Mandel and Métais [21], cell-free DNA (cfDNA) is increasingly used as a biomarker for a wide range of clinical indications, from non-invasive prenatal testing (NIPT) to sepsis and cancer [22]. CfDNA has proven to be a highly suitable and dynamic biomarker for pathogen identification in sepsis [23]. Accordingly, Grumaz et al. could show that cfDNA sequencing using next-generation sequencing (NGS) resulted in a six-times higher sensitivity for pathogen identification than blood culture, with an expert plausibility of 96% [24]. However, NGS is still associated with limitations regarding costs per sample, high upfront costs for sequencing devices, and a lack of AMR detection. In contrast, Nanopore sequencing is characterized by low to moderate upfront costs, immediate processing, and real-time analysis, as well as high flexibility for testing due to small size [25,26]. This enables new diagnostic possibilities, from rapid outbreak surveillance to infection diagnostics and AMR profiling [27,28,29]. Nonetheless, sepsis diagnostic based on Nanopore sequencing remains challenging and only a few studies are reported but without the usage of cfDNA as the biomarker of choice [30]. Previously, a workflow for unbiased, real-time cfDNA-sequencing on septic samples was established, which could identify pathogen burden within the first hours of sequencing [31]. The extrapolation of results could show that over 90% of pathogens could be detected with cfDNA Nanopore sequencing. Still, this approach was only applicable to samples with a high pathogen burden while also failing to detect AMRs.

With SUPSETS (suppression PCR-based selective enrichment sequencing), we aimed to establish a diagnostic platform based on a megaplex, highly specific two-step suppression PCR and subsequent Nanopore sequencing to detect the most common sepsis-causative pathogens across three kingdoms and main AMR genes.

## 2. Results

### 2.1. SUPSETS Procedure

**Figure 1:**
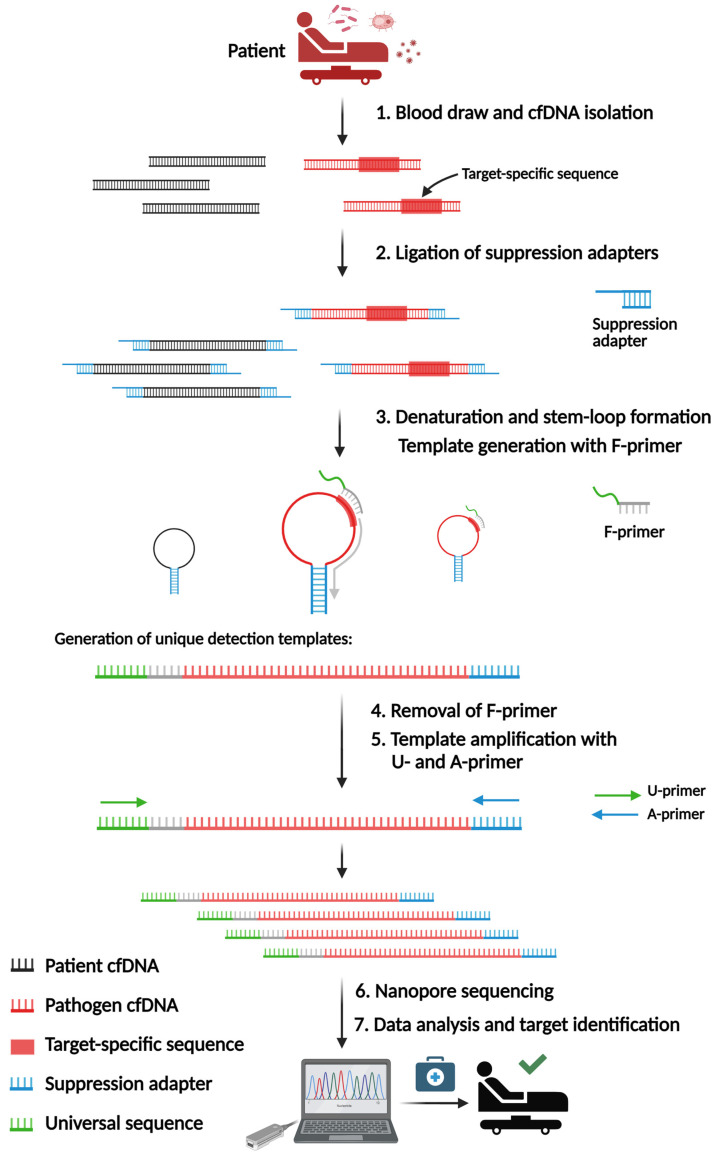
Principle and workflow of SUPSETS: from blood draw and cfDNA isolation (step 1), suppression adapter ligation (step 2), and multiplex suppression PCR (steps 3–5) to sequencing and data analysis for pathogen and AMR detection (steps 6 and 7). Image created by BioRender.

SUPSETS combines the selective amplification of target sequences with subsequent next-generation sequencing on randomly fragmented cell-free DNA (cfDNA). We identified species-specific sequences for the most common sepsis-causative pathogens and AMRs. After a blood draw, cfDNA is isolated from plasma followed by ligation of suppression adapters to all cfDNA fragments (Figure 1). The GC-rich adapter sequence forces self-annealing, leading to a stem-loop formation upon denaturation, which diminishes unwanted amplification [32,33]. Following adapter ligation, F-primers are used for the initial elongation of a fragment, forming a detection template after hybridization to its target sequence. F-primers consist of a universal sequence (US) [34] and a target-specific region at the 3′ end. For n targets, n F-primers are used to minimize primer amount, which enables higher multiplexing. Elongated cfDNA fragments (detection templates) are flanked by the 5′ universal sequence and the 3′ adapter sequence, allowing an amplification of all detection templates with the same primer pair (US-FW and Ad-rev). Amplified detection templates undergo library preparation for Nanopore sequencing, followed by data analysis for species detection, either after the finished run or in real-time, by mapping reads to a target amplicon database.

Candidate primers were tested to determine the most specific and efficient primers (Supplementary Figure S1A–D). Exemplary for HSV I, primer candidates were finally tested with 4000 GE digested and adapter-ligated HSV I DNA (Supplementary Figure S1D). To mimic patient samples, 6000 GE of digested and adapter-ligated human DNA was spiked in. Primers producing unspecific signals were discarded in favor of HSV I T1 and HSV I T2 primers, which produced strong signals with little background (Supplementary Figure S1C,D).

**Figure 2:**
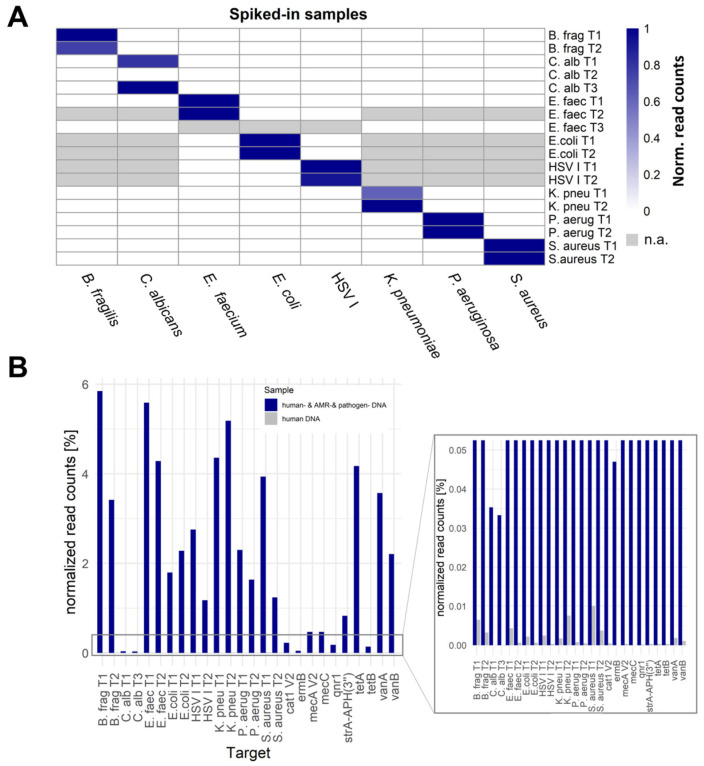
SUPSETS technical validation on artificial spike-in samples. (**A**) Technical validation of SUPSETS with single spike-in samples: 4000 GE digested and adapter-ligated genomic pathogen DNA was spiked in 6000 GE digested and adapter-ligated genomic human DNA, and the SUPSETS workflow was performed with a primer mix containing two primers per tested targeted pathogen, one genus-specific *Candida* primer, and one general 16 S-targeting primer. Single primers were exchanged between experiments to improve performance or added with the expansion of the targeted pathogen panel (Supplementary Table S1). X-axis: Spike-in pathogen per column. Y-axis: Regions targeted by pathogen-specific primers. (**B**) Reads per primer-related amplicon region for artificial human DNA sample with and without spiked-in target DNA: 4000 GE digested and adapter-ligated genomic DNA for each pathogen and AMR was spiked to 6000 GE digested and adapter-ligated genomic human DNA in the human- & AMR- & pathogen DNA sample, and incubated with a primer mix containing two primers per targeted pathogen, one genus-specific *Candida* primer, one general 16 S-targeting primer, and sixteen primers targeting genes for antimicrobial resistances. For negative control, no pathogen-derived DNA was included in the same experimental setup, only using 6000 GE digested and adapter-ligated human DNA. The X-axis shows the target regions excluding primers C. alb T2, AAC(6)-Ib, TEM116, ndm1, OXA48, blaCTX-M-15 V2, and blaKPC-2 V2 due to performance lack. The Y-axis depicts the percentage of normalized read counts. For the corresponding primers, see Supplementary Table S1. n.a., not available. Normalized counts = max(coverage)_i_/(Σ_(i→j)_max(coverage)_i_ + Σhuman reads); column corrected. i for target region.

In general, F-primers were tested for their performance in a spike-in experiment, where 4000 GE digested and adapter-ligated DNA of each pathogen or AMR plasmid was spiked in 6000 GE digested and adapter-ligated human DNA. A detailed list of used primers per experiment is summarized in Supplementary Table S1. Approximately 72.2% (SD 15.92%) of reads were of human origin, depending on the spike-in input (Supplementary Table S2). Dependent on the respective pathogen, the number of detected amplicon reads differed from 258 reads (~0.04% of all filtered reads per sample) for *C. albicans* amplicon T3 to 297,783 reads (~9.13% of all filtered reads per sample) for *P. aeruginosa* amplicon T2. All target amplicons could be discriminated from background, with the exception for *C. albicans* amplicon T2 (Figure 2A). The highest proportion of amplicon reads in contrast to all filtered reads was shown for B. frag T1 (~11.67% of all filtered reads per sample).

To show the feasibility of the complex panel in a multiple-pathogen sample, all addressed pathogens and AMRs were spiked into human DNA and SUPSETS was performed with a combined primer panel (=sample “human- & AMR- & pathogen DNA”; for the used primers, see Supplementary Table S1). As a negative control, human DNA without any other pathogen spike-in was tested with the same primer mix (sample “human DNA”). All detected amplicons could be clearly distinguished between both samples (Figure 2B). The detected amounts of on-target reads exceeded the human DNA-only sample by at least two orders of magnitude (*S. aureus* T2) and by up to three orders of magnitude for most other amplicons. However, primers C. alb T2, AAC(6)-Ib, TEM116, ndm1, OXA48, blaCTX-M-15 V2, and blaKPC-2 V2 could not be detected.

### 2.2. SUPSETS Validation on Selected Clinical Sepsis Samples

**Figure 3:**
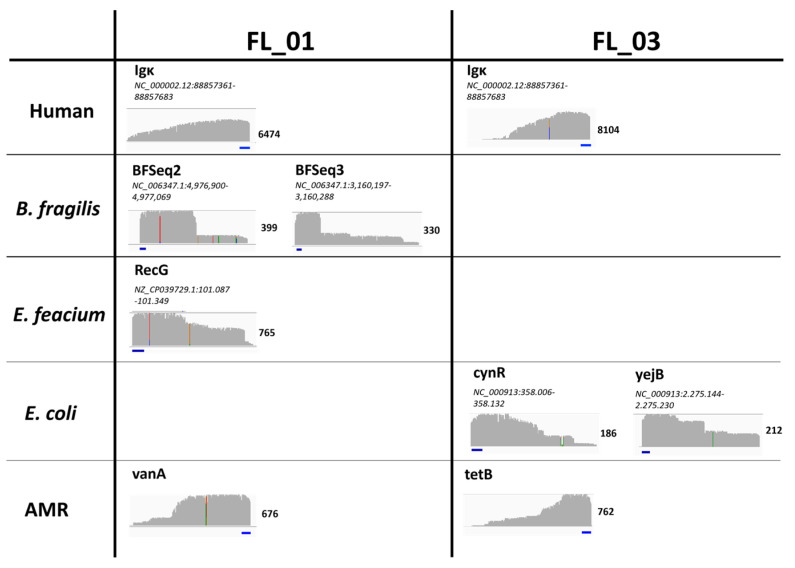
Appearance of SUPSETS-detected cfDNA amplicons. The results of SUPSETS workflow for clinical samples FL_01 and FL_03 were mapped against the respective pathogen or AMR reference genome and visualized in IGV browser. For each amplicon, maximum coverages with a coverage greater than two were counted and visualized. The primer binding site is depicted in blue. Mismatches are displayed in multi-color.

For a first proof-of-concept clinical validation, selected septic samples from Grumaz et al. (2019) [24] were used as a reference for pathogen detection (Supplementary Table S3). To see the cfDNA amplicon profiles of these samples, the sequencing results were mapped to the respective pathogen and AMR reference genome. Exemplarily shown for samples FL_01 and FL_03, no distinct reads could be detected in off-target regions, and clear signal peaks were found in the selected regions, which started with the primer sequence (Figure 3). While the beginning of the amplicon at the 5′ end is clearly defined by the primer, there is no clear terminus at the 3′ end. This reflects the randomly fragmented nature of cfDNA, resulting in cfDNA fragments of different length and therefore undefined primer positions of the target region within the fragment (Figure 3).

**Figure 4:**
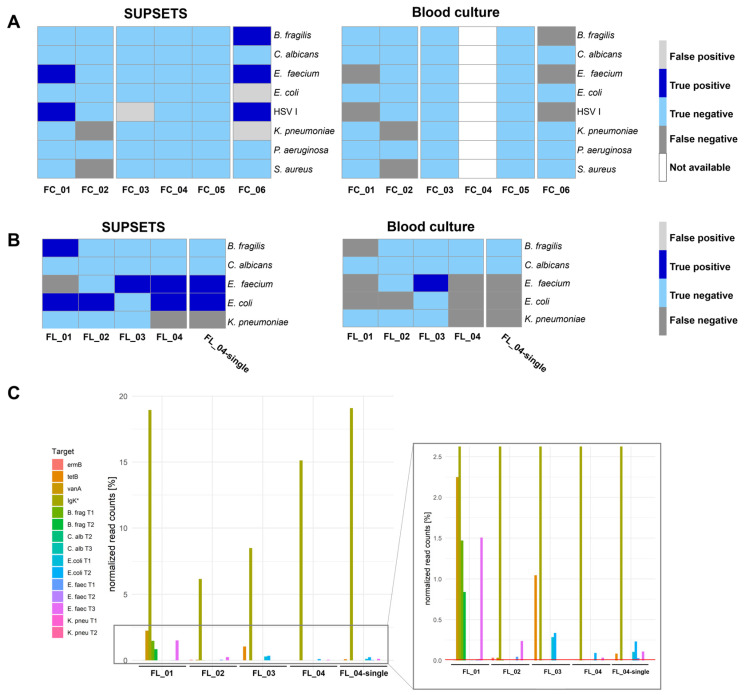
Validation on selected clinical samples. Septic patient samples were selected according to their pathogen and AMR profile from the MIRSI study [21] and applied to the SUPSETS workflow. (**A**) Qualitative analysis comparing NGS data (“ground truth”) to the SUPSETS experiment on MinION Flow Cell and blood culture results. (**B**) Qualitative analysis comparing NGS data (“ground truth”) to the SUPSETS experiment on MinION Flongle and blood culture results. (**C**) Processed reads were normalized to filtered mapped reads (Q-score > 9) for each sample for four selected patient samples within one MinION Flongle sequencing experiment. Samples FL_01–04 were sequenced together on one MinION Flongle run for 24 h; FL_04-single indicates a single-sample MinION Flongle sequencing experiment for 3 h. The red vertical indicates the threshold of 0.01% normalized reads. Target amplicons are indicated according to the color legends. (**A**,**B**) True positive hits compared to NGS data are indicated in dark blue, false positive signals in light grey, light blue reflects true negative and dark grey false negative results, and white is for no data available. * Igκ reads were taken after mapping against the reference genome (hg38).

Samples were analyzed with different primer panels using either a MinION Flongle accompanied with lower sequencing throughput or applied to a MinION Flow Cell (Figure 4). For the MinION Flow Cell samples, a positive signal was considered if at least one read was detected for at least one of the two pathogen-targeting primers. Five out of seven pathogens were detected correctly as positive by SUPSETS; in contrast, none were identified by blood culture (Figure 4A and Supplementary Table S4). Among all pathogen-containing samples (FC_01, FC_02, and FC_06), three false positive results were obtained, with none for blood culture. In negative controls (FC_03 to FC_05), comprising post operative samples and healthy controls, one false positive result in sample FC_03 (Figure 4A) could be detected. Accordingly, the best performance was found for *E. faecium*- and *B. fragilis*-targeting primers with exclusively correct positive and negative findings.

For MinION Flongle experiments, four clinical samples were run in one experiment (FL_01–04), with sample FL_04 tested additionally as a solitary sample (FL_04-single). Overall, SUPSETS detected six out of eight pathogens correctly (Figure 4B and Supplementary Table S5), with at least two reads and 0.01% of all filtered reads considered as a positive result. No false positive signal was obtained and every negative control sample from clinical metagenomics was confirmed as negative. In the two samples with multi-pathogen burden, FL_01 and FL_04, two out of three pathogens were detected, resulting in two false negative results. In comparison, only one of eight pathogens was detected correctly as positive in blood culture (Figure 4B).

For samples FL_01–04, the internal sequencing and PCR control Igκ was used to target human cfDNA (Figure 4C). Human amplicon Igκ received most of the normalized reads, ranging between 6.2% and 19.1% of total reads. Amplicons targeting the same pathogen do not necessarily result in a comparable percentage of read counts. Exemplarily for sample FL_01, roughly 2.24% of read counts were received for B. frag T1 and T2, on the one hand. On the other hand, for *E. faecium* reflecting target regions, only the amplicon E. faec T3 could be detected above the set threshold with 1.5%. Interestingly, for patient FL_04, differences could be identified regarding signal intensity and results when analyzed as a single sample (Figure 4C). While the multiplex sample FL_04 was not tested positive for both *E. faecium* and *E. coli* amplicons, FL_4-single had a three-times higher percentage of normalized read counts, e.g., for E. faec T3 from 0.0034% to 0.0109%.

AMRs could not be detected in the clinical metagenomic reference analysis from [24]. However, in SUPSETS, several AMRs were detected in selected clinical samples (Figure 4C and Table 1). The SUPSETS analysis revealed a potential resistance for tetracycline (tetB) in patients FL_03 and FL_04-single, which was not documented for the corresponding patients before. A vancomycin resistant *E. faecium* (VRE) was detected for patients FL_01, FL_02, and FC_06. In all these samples, vancomycin resistance was confirmed through the results of other clinical specimens including surgical, abdominal, or wound swabs. In patient FL_02, macrolide resistance against gene ermB was additionally detected. By means of blood culture analysis, no AMRs could be detected due to negative blood culture results.

### 2.3. First Read Detection of SUPSETS within Selected Sepsis Samples

**Figure 5:**
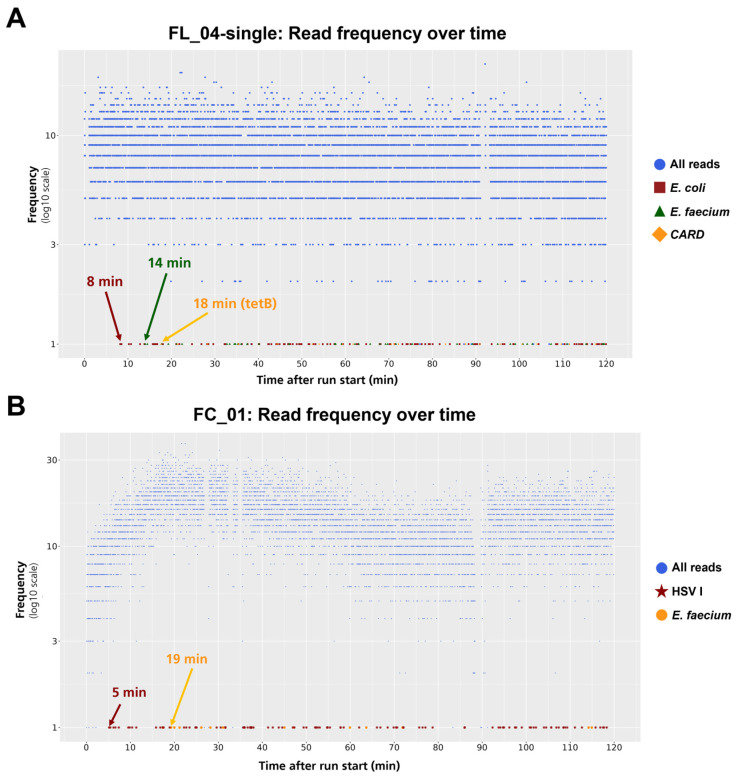
Real-time detection of first pathogen and AMR reads. For a retrospective analysis, processed reads are shown at the time they were sequenced. Analysis is shown for two different clinical samples: (**A**) (FL_04-single) and (**B**) (FC_01). Arrows with time data indicate the first target hits according to the shape and colored legend, respectively.

The outcomes of septic patients improve when early and targeted therapy is administered, requiring the early detection of pathogens [14]. Therefore, we retrospectively investigated at which point in time the first amplicon read corresponding to a pathogen or AMR was detected. Clinical samples FL_04-single and FC_01 were selected for the retrospective analysis (Figure 5). Remarkably, for sample FL_04-single, all three expected species and AMR targets (*E. coli*, *E. faecium*, and tetB) could be detected within the first 18 min of sequencing, with both pathogens’ first signals detected after 8 min for *E. coli* and 14 min for *E. faecium* (Figure 5A). Comparable findings were revealed for patient FC_01, where HSV I was first detected after 5 min and *E. faecium* after 19 min (Figure 5B). Therefore, the early detection of pathogenic and AMR burden with real-time analysis is feasible and might reduce the time-to-diagnosis.

## 3. Discussion

Sepsis and antimicrobial resistances (AMRs) have severe health implications, due to inadequate diagnostic options. Since blood culture has severe limitations, several approaches like clinical metagenomics on cfDNA for the identification of pathogens have been published [24,35,36]. Based on these promising results, we aimed for a targeted and more rapid and cost-effective approach than unbiased clinical metagenomics. Since cfDNA is a challenging molecule due to its random fragmentation, low concentration, and short length (Figure 3), we established SUPSETS, a megaplex suppression PCR-based selective target sequencing for pathogen and antimicrobial resistance detection on cell-free DNA in sepsis (Figure 1).

We first demonstrated the feasibility of SUPSETS on artificial samples, which revealed high discriminatory power between target regions. Using SUPSETS, we could show that even in a multi-pathogen background comprising eight different pathogens, all pathogens could be resolved (Figure 2B) in contrast to blood culture analysis [31,37].

Blood culture is already known to be limited with respect to pathogen detection in sepsis. Grumaz et al. showed that, in their study, over the full 28-day period, 71% of samples were positive with clinical metagenomics and only 11% with blood culture, with an expert plausibility of 96% [24]. We therefore benchmarked our results against clinical metagenomics using samples from this study. With these selected clinical samples, SUPSETS clearly outperformed blood culture results, with 11 of 15 correctly detected pathogens (73.3%) compared to only 1 correctly detected pathogen with blood culture (6.7%). SUPSETS displayed three false positive results in Flow Cell experiments, resulting in a specificity of 94.3% (50 of 53 truly negative results) (Figure 4A,B). Molecular approaches like SepsiTest reveals a sensitivity and specificity of 66.7 and 94.4% compared to blood culture, respectively, with an additional 15 positive results for SepsiTest and only 10 results positive in blood cultures [38].

Clinical metagenomics cannot reliably detect AMRs, as sequencing depth in most cases is too low [24,31]. Using SUPSETS, we could detect several AMRs in clinical samples in contrast to blood culture. The results were additionally supported by other clinical specimens for vancomycin resistance or for ermB as an intrinsic AMR in *E. faecium* [39]. Resistance findings by SUPSETS might have clinical impact; for instance, patient S10 (FL_02) was treated with vancomycin while the status and microbial load were worsening, due to a non-diagnosed VRE infection [24]. Our results provided evidence for the adaptation of treatment that might have led to an earlier and more targeted treatment.

Early adequate treatment is crucial in sepsis [40]. Blood culture analysis takes up to several days for a potentially positive result, while clinical metagenomics delivers results in up to 48 h. Within SUPSETS, targeted amplification and Nanopore sequencing allow real-time analysis to reduce the time-to-analysis. The retrospective analysis of clinical samples shows that the first pathogen and AMR-assigned reads were detected within a few minutes (Figure 5). Mapping to a database containing only the selected target sequences after basecalling would accelerate the SUPSETS’ procedure in contrast to conventional NGS approaches. Several approaches in the literature have already demonstrated the feasibility of real-time analysis [41,42]. A SUPSETS real-time detection of pathogens and AMRs would reduce time-to-diagnosis to approximately 12 h and enable the earlier targeted therapy of septic patients on the same day (Supplementary Table S6). Protocol optimization could lead to a further decrease in detection time.

Although blood culture is currently much more affordable, study results from clinical metagenomic studies clearly show the advantages of sequencing in terms of time savings and sensitivity [24,37]. Applying SUPSETS, we decreased the costs of clinical metagenomics to an estimated EUR 160–225 per sample. Also, investments are moderate and therefore in-house analysis using SUPSETS seems manageable (Supplementary Table S7). With significantly high costs for a septic patient per day at ICU and more reliable results than blood culture (Figure 4), SUPSETS might be an attractive option with respect to cost savings [7,43].

SUPSETS also exhibits the limitations of a targeted approach. Since only selected targets are addressed, pathogens not included into the panel cannot be detected. Since only species-specific primers are required, the tested panel can be quickly expanded and adapted to other targets. Other pathogen and AMR targets should be re-evaluated (OXA-48, KPC-2, ndm-1) and included to increase clinical value. Recently, Oxford Nanopore released information that their chemistry might be sensitive to light, which might have led to decrease in output and quality, particularly for short reads. Since this chemistry was used for Flow Cell experiments, this might explain performance differences between those and Flongle experiments [44]. 

## 4. Materials and Methods

### 4.1. Ethics Approval and Consent to Participate in Clinical Samples

Septic patient samples from a previously published clinical study were used [24]. The monocentric clinical study (S-097/2013) was conducted in the surgical intensive care unit of Heidelberg University Hospital, Germany (German Clinical Trials Register: DRKS00005463); with study and control patients or their legal representatives signing written informed consent. Results from this previous study were used as a reference for the clinical validation of the SUPSETS experimental research study. All study procedures were approved by the local ethics committee (Ethics Committee of the Medical Faculty of Heidelberg—Trial Code No. S-097/2013). Blood from healthy individuals was acquired commercially from Biomex (Heidelberg, Germany).

### 4.2. Microbiology and Preparation of Microbial DNA

Frozen samples of bacteria (*Enterococcus faecium* DSM 20477, *Klebsiella pneumoniae* DSM 30104, *Bacteroides fragilis* DSM 2151, *Pseudomonas aeruginosa* DSM 50071 and *Staphylococcus aureus* DSM 20231) and *Candida albicans* SC5314 (ATCC MYA-2876) were thawed on ice. A total of 50 µL of each pathogen solution was transferred to 10 mL of prewarmed Brain Heart Infusion Broth (Merck Millipore, Darmstadt, Germany). After incubating overnight at 37 °C and 150 rpm, microbial DNA extraction was performed with a PureLink Genomic DNA kit (Invitrogen, Waltham, MA, USA), according to the manufacturer’s advice. Concentration was checked with a Qubit 3.0 fluorometer dsDNA High Sensitivity (HS) assay kit (Thermo Fisher, Waltham, MA, USA) and quality-controlled with a DNF-488 High Sensitivity Genomic DNA Analysis kit (Fragment Analyzer Automated CE, Agilent Technologies, Santa Clara, CA, USA). Isolated genomic DNA for *Escherichia coli* strain B and human (both Sigma-Aldrich, Steinheim, Germany) were bought and HSV I was provided by Dr. Florian Full. The genome sequences of 16 AMRs were taken from the Comprehensive Antibiotic Resistance Database (CARD) [45], reduced to the relevant regions, and combined with a DNA linker and a restriction site in one DNA cassette each. These cassettes were combined to four plasmids and ordered at Integrated DNA Technologies GmbH (Munich, Germany) (Supplementary Figure S2). To mimic cfDNA in length, pathogen DNA was digested with the restriction enzymes AluI and HpyCH4V (both New England Biolabs, Frankfurt, Germany), according to the manufacturer’s protocol. The digested DNA was purified with 1.8× AMPure XP beads (Beckman-Coulter, Pasadena, CA, USA), according to the manufacturer’s advice. DNA concentration was checked with a Qubit 3.0 fluorometer dsDNA High Sensitivity (HS) assay kit and quality-controlled with a DNF-474 High Sensitivity NGS Kit (Fragment Analyzer Automated CE, Agilent Technologies, Santa Clara, CA, USA).

### 4.3. Identification of Species- or Genus-Specific Nucleotide Sequences and Primer Design

For the identification of species-specific nucleotide sequences, the NCBI reference genome of the pathogen of choice (Supplementary Table S8), excluding plasmids, was used to generate 10 million Illumina sequencing reads using InSilicoSeq (-model HiSeq; v1.5.0) [46]. These simulated sequencing reads were taxonomically classified using Kraken with the standard prebuilt reference database (v2.1.2) [47]. Only classified reads with a higher confidence score than 0.95 for the pathogen of choice were chosen for further use. A total of 1000 reads were randomly selected and blasted against an NCBI NR database excluding the pathogen of choice using blastn (-num_alignments 10-word_size 20-task blastn -short -evalue 1e-50-negative_taxids <pathogenTAXID>; v2.13.0) [48,49]. Reads with a match to any other organism were discarded.

For the identification of genus-specific nucleotide sequences, the methodology used previously for species-specific sequences was slightly altered. The initially simulated Illumina sequencing reads were based on the reference genomes of all species belonging to the genus of interest, based on the NCBI taxonomy database (defined with the ‘get_species_taxids’ script from the blast tools). In the blastn analysis of the 1000 selected nucleotide sequences, the E-value threshold was reduced to 1e-20.

Using Primer3 (libprimer3 release 2.6.1), primers were generated for the identified species-specific or genus-specific nucleotide sequences with the following settings: a minimum amplicon length 50 bp, a primer length between 18 to 22 bp, a maximum of two repeating bases, no unknown bases (N), a melting temperature between 59 °C and 64 °C, and a GC content between 40% and 60% [50]. The generated primers were validated against the genus-specific nucleic acid database using local BLAST (using “-taxidlist” blastn option) to ensure specificity for defined TaxIDs. Validation also included a BLAST search against the nt database to exclude hits on non-target genomes. Furthermore, the extracted primers were blasted to confirm specificity to the desired genus without hits on other microbial or human sequences within the top hundred hits. Selected primer sequences were ordered at Sigma-Aldrich.

### 4.4. One-Step PCR for Primer Testing

For the evaluation of suitable primers, a one-step PCR with putative US-target-primers was performed. A total of 40,000 genome equivalents (GE) of digested, adapter-ligated pathogen DNA was mixed with 1 µL dNTP mix (10 mM -Sigma-Aldrich, Steinheim, Germany), 2.5 µL 10× AmpliTaq Gold 360 buffer, 2.5 µL magnesium chloride (25 mM), 0.25 µL AmpliTaq Gold 360 DNA polymerase (all Thermo Fisher, Waltham, MA, USA), 1 µL unique US-target-primer (0.2 µM), and 1 µL Ad-rev primer (0.2 µM) (both Sigma-Aldrich, Steinheim, Germany), and filled with nuclease-free water up to 25 µL. After mixing, the reaction was incubated for 4 min at 95 °C, followed by 38 cycles of 95 °C for 20 s, 65 °C for 30 s and 72 °C for 30 s. Quality control was performed as described above.

GE were calculated according to the following formula:

(1) $$m\left[pg\right]=\frac{genome\;size\left[bp\right]\cdot M_{w}\left[\frac{g}{mol}\right]\cdot GE\cdot 10^{12}[\frac{pg}{g}]}{N\left[\frac{molecules\;or\;bp}{mol}\right]}=\frac{genome\;size\cdot 650\cdot GE\cdot 10^{12}}{6.022\cdot 10^{23}}$$

### 4.5. SUPSETS Workflow

Suppression adapters were generated from a long (Adapter_long) and short (Adapter_short) oligonucleotide (Sigma-Aldrich, Steinheim, Germany), using sequences first published by Matz et al. (1997) [51]. For hybridization, 20 µL of both oligonucleotides was incubated with 10 µL 10× T4 ligase mix (New England Biolabs, Frankfurt, Germany) and 50 µL nuclease-free water for 3 min at 95 °C and cooled down overnight at RT.

The SUPSETS workflow starts with ligating suppression adapters to blunt-end DNA fragments, either prepared cfDNA or digested genomic DNA. A total of 20 µM suppression adapter was added at a ratio of 1:20 to DNA (600–700 ng) together with 400 U of T4 ligase and 10× T4 ligase buffer, adjusted with nuclease-free water to reach a one-time concentration of ligase buffer. The reaction was incubated for 2 h at RT or 16 °C overnight. After heat inactivation at 65 °C for 10 min, adapter-ligated DNA was purified with 1.6× AMPure XP beads (Beckman Coulter, Pasadena, CA, USA) and the concentration and profile validated as described above.

For elongation, 2.5 µL 10× AmpliTaq Gold 360 buffer was mixed with 2.5 µL magnesium chloride (25 mM), 1 µL dNTP mix (10 mM—Sigma Aldrich), 0.25 µL AmpliTaq Gold 360 DNA polymerase, and a panel of Fusion primers (F-primers), with a final concentration of 0.3 µM per F-primer. The list of F-primers and used F-primers per experiment can be found in Supplementary Tables S9 and S1, respectively. As input, either 40,000 to 4000 and 6000 genome equivalents of adapter-ligated, digested pathogen or human DNA, respectively, was used and the reaction filled up to 25 µL with nuclease-free water (Equation (1)). For clinical samples, a defined volume of 15.92 µL blunt-end cfDNA was used. The reaction was incubated for 5 min at 95 °C, followed by 66 °C for 20 min and 72 °C for 5 min. The elongation product was purified with 1.6× AMPure XP beads to remove excessive F-primers and used for amplification. A total of 13.5 µL of this elongation product was mixed with 2.5 µL 10× AmpliTaq Gold 360 buffer, 2.5 µL magnesium chloride (25 mM), 1 µL dNTP mix, 4.25 µL nuclease-free water, 0.25 µL AmpliTaq Gold 360 DNA polymerase, and US-FW and Ad-rev primers (both Sigma-Aldrich, Steinheim, Germany), each with a final concentration of 0.2 µM. The reaction was incubated for 4 min at 95 °C, followed by 38 cycles of 95 °C for 20 s, 65 °C for 30 s, and 72 °C for 30 s, and a final elongation of 5 min at 72 °C. The amplification product was purified with 1.6× AMPure XP beads and quality control performed as described above.

Purified amplification products were applied to Nanopore sequencing. For MinION Flow Cell experiments, the Native Barcoding Kit 24 V14 (Oxford Nanopore Technologies, ONT, London, UK) was used according to the manufacturer’s advice. For MinION Flongle experiments, the Ligation Sequencing Kit SQK-LSK 109 for sample preparation and the Native Barcoding Expansion Kit EXP-NBD 104 were used according to the manufacturer’s protocol. For the end-prep reaction, 12.5 µL of sample was used as input. After the barcoding and pooling of the reaction, native adapter was ligated with 5 µL T4 Ligase. A MinION Flow Cell (R.10.4.1) or Flongle (R.9.4.1) was primed and the DNA sequencing library loaded according to the protocol. Samples for technical validation were sequenced for a total of 24 h. Clinical samples were sequenced as follows: FL_01–04 for 24 h, FL_04-single for 3 h, and FC_01–06 for 72 h.

### 4.6. Analysis of Sequencing Results

After sequencing with MinION Flongle, the basecalling and demultiplexing of sequencing data were performed with Guppy (version 6.5.7 ONT), using the high accuracy model with a minimal allowed Phred score equal to 9 (dna_r10.4.1_e8.2_400bps_5khz_hac). Afterwards, adapters and barcodes were trimmed using qcat [49], followed by quality control using Nanoplot [52]. Next, quality score-based read filtering was performed with a minimum q-score of nine and a minimum length of 50 bp with NanoFilt [52]. For results represented in Figure 3, quality-checked reads were mapped to the corresponding reference genomes using mimimap2 with default settings [53]. For the antibiotic resistances, an artificial mapping reference was generated, where all available AMR nucleotide sequences from CARD were concatenated to a fasta file. Reads with a mapq score below 20 were removed from the mapping output using samtools [54]. Sorted and indexed bam files were then visualized by IGV Browser and exemplarily presented in Figure 3 (v2.16.1) [55]. For MinION Flow Cell and further analysis of MinION Flongle experiments in Figure 2 and Figure 4, basecalling, demultiplexing, adapter trimming, quality control, and quality filtering were performed as described above. Only reads with a q-score of at least 12 (9 for Flongle) and a size range between 100 to 1000 bp were kept. Mapping was performed with minimap2 against an artificial mapping reference consisting of the previously identified target regions for evaluated pathogens, including AMRs and the human genome (hg38) [53]. Data obtained using the SUPSETS workflow were evaluated in the context of Illumina-based, clinical metagenomics results previously published [24].

For the retrospective analysis of first received amplicon reads, timestamps for every barcode from Guppy-generated, filtered reads were extracted. (1) Filtered reads were mapped to a reference pathogen genome or to the artificially generated AMR reference using minimap2. (2) Only uniquely-mapped reads and corresponding read-ID were extracted using samtools. (3) The two generated lists were combined to find timestamps of uniquely-mapped reads using a custom perl script. After repeating the described steps 1–3 for every reference genome, results were plotted using a custom R script.

### 4.7. Clinical Sample Preparation

Septic samples and isolated cfDNA were obtained from a previously published study (Supplementary Table S3) [24]. For the ligation of suppression adapters, cfDNA was prepared with the NEB Quick Blunting Kit (New England Biolabs, Frankfurt, Germany), according to the manufacturer’s protocol, with 19 µL of cfDNA as input.

## 5. Conclusions

Sepsis and antimicrobial resistances (AMRs) are major global health threats, also due to a lack of appropriate clinical diagnostic tools. Within SUPSETS, we established a platform to bridge the current diagnostic gap by combining a multiplex suppression PCR with subsequent Nanopore sequencing to detect sepsis pathogens and AMRs. We could show the potential by means of the simultaneous and specific detection of up to eight different pathogens and ten AMRs. A first proof on selected clinical samples outperformed blood culture analysis and delivered comparable results to clinical metagenomic analysis. We could show that SUPSETS can detect pathogenic and AMR signals within minutes after sequencing, opening the field for real-time sequencing analysis to decrease time-to-diagnosis further. Taken together, SUPSETS shows great potential in pathogen and AMR detection in septic patients. Further studies on clinical samples in sepsis or related fields like cancer panel diagnostics or environmental sample assessment have to be carried out to evaluate SUPSETS. Nonetheless, the obtained results suggest that SUPSETS as a diagnostic platform might be a valuable diagnostic in-house solution for the future of sepsis/AMR and related clinical fields.

## Figures and Tables

**Table 1:** Antimicrobial resistances detected in clinical samples. Resistance genes found with SUPSETS are indicated. Corresponding information about the resistome for blood culture and the analysis of other clinical specimens from the patients are derived from Grumaz et al. (2019) [21]. - resistance or resistant phenotype not detected, ~ no associated clinical samples available.

Sample	SUPSETS	Blood Culture	Clinical Specimen
FC_06	vanA	-	Vancomycin
FL_01	vanA	-	Vancomycin
FL_02	vanA, ermB	-	Vancomycin
FL_03	tetB	-	~
FL_04-single	tetB	-	~

## Data Availability

Original patient data are unavailable due to ethical considerations; only pseudonymous information was used. The original data presented in the study are openly available in NCBI at https://www.ncbi.nlm.nih.gov/bioproject/PRJNA1100967.

## Supplementary Materials

The following supporting information can be downloaded at: https://www.mdpi.com/article/10.3390/ijms25105463/s1.
