# Supplementary material for: Suppression PCR-Based Selective Enrichment Sequencing for Pathogen and Antimicrobial Resistance Detection on Cell-Free DNA in Sepsis—A Targeted, Blood Culture-Independent Approach for Rapid Pathogen and Resistance Diagnostics in Septic Patients
Source: Int J Mol Sci. 2024 May 17;25(10):5463. doi: 10.3390/ijms25105463 (PMC11121775; doi:10.3390/ijms25105463)
Supplement: Supplementary file 1 [file ijms-25-05463-s001.zip › ijms-2996825-supplementary.pdf]

Article

Suppression PCR-based selective enrichment sequencing for pathogen and antimicrobial resistance detection on cell-free DNA in sepsis - A targeted, blood culture independent approach for rapid pathogen and resistance diagnostics in septic patients

Supplement

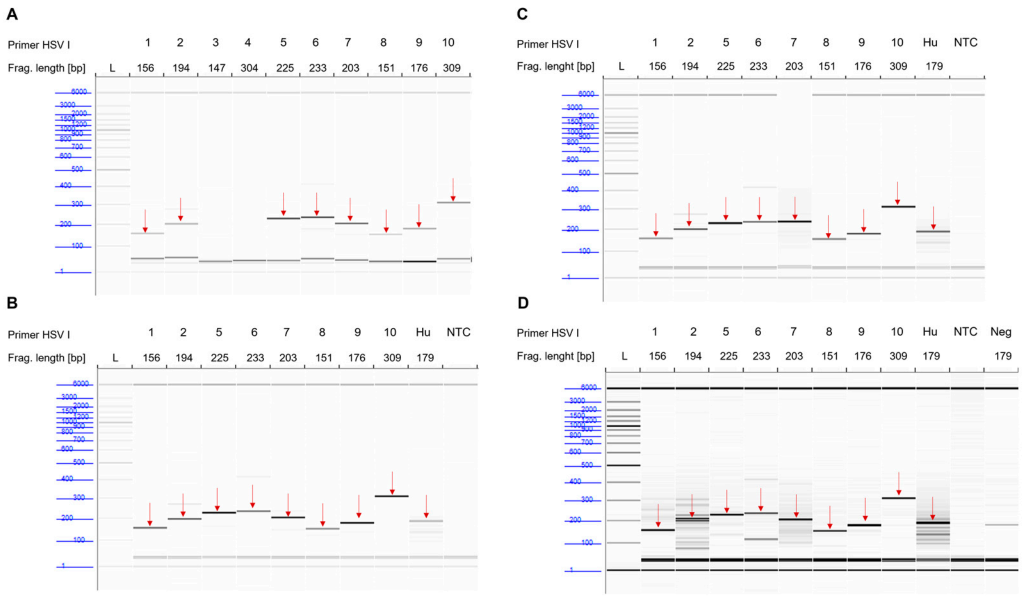

Supplement Figure S1. Identification of suitable HSV I primers. For the identification of most suitable SUPSETS target regions, bioinformatically identified primers were tested. Each lane represents a PCR product with the indicated HSV I primer. For all PCR experiments, the expected fragment length in bp is depicted and inside the gel image displayed with a red arrow. A: 40,000 GE of digested, adapter ligated HSV I DNA were tested in a one-step PCR as described in methods section. B: 40,000 GE digested, adapter ligated HSV I DNA were tested by the complete SUPSETS workflow. C: 40000 GE digested, adapter ligated HSV I DNA were spiked in 6,000 GE human digested, adapter ligated DNA and tested by the complete SUPSETS workflow. D: 4,000 GE digested, adapter ligated HSV I DNA were spiked in 6,000 GE human digested, adapter ligated DNA and tested by the complete SUPSETS workflow. NTC, non-template control; Hu, human only sample; Neg, HSV I DNA with human primer.

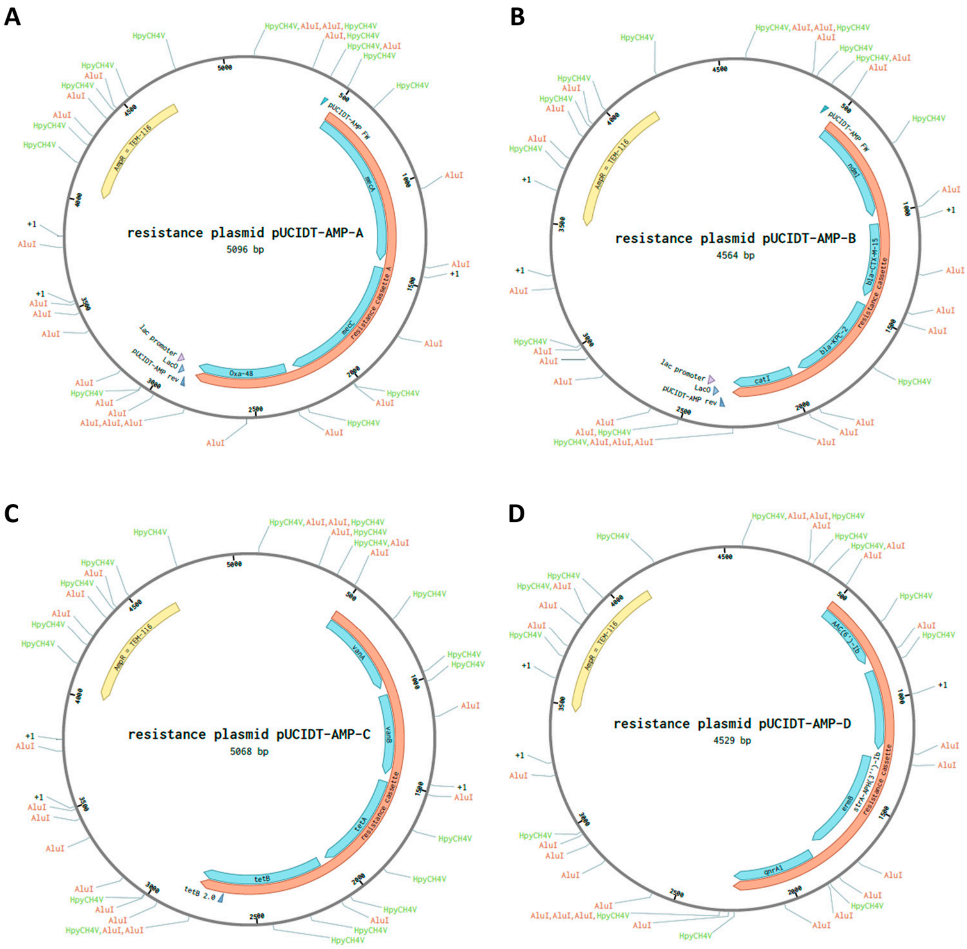

Supplement Figure S2. Antibiotic resistance plasmids. Antibiotic resistance plasmids. Resistance cassettes with relevant parts of AMR genes were cloned in the plasmid pUCIDT-AMP by Integrated DNA Technologies GmbH (IDT). A: Resistance plasmid A contains parts of the resistance genes mecA, mecC and Oxa-48. B: Resistance plasmid B contains parts of the resistance genes ndm-1, bla-CTX-M-15, bla-KPC-2 and catI. C: Resistance plasmid C contains parts of the resistance genes vanA, vanB, tetA and tetB. D: Resistance plasmid D contains parts of the resistance genes AAC(6')-Ib, strA-APH(3'')-Ib, ermB and qnrA1. All resistance cassettes are indicated in orange, while the resistance gene parts are depicted in blue. The selecting TEM116 ampicillin resistance is depicted in yellow. Furthermore, the used restriction enzymes AluI and HpyCH4V are shown. Images created in benchling.com.

Supplement table S1. Primer set used per experiment. Spike-In A: Spike-In *E. faecium*, *E. coli*, and HSV I. Spike-In B: Spike-In *B. fragilis*, *C. albicans*, *K. pneumoniae*, *P. aeruginosa* and *S. aureus*.

	Technical validation			HSV I testing		Clinical samples	
	**Spike-In A**	**Spike-In B**	**Multiplex**	**1 Step**	**SUPSETS**	**Flongle**	**Flow Cell**
B. fragilis	B. frag T1 & T2	B. frag T1 & T2	B. frag T1 & T2	-	-	B. frag T1 & T2	B. frag T1 & T2
C. albicans	C. alb T1 – T3	C. alb T1 – T3	C. alb T1 – T3	-	-	C. alb T1 & T3	C. alb T1 – T3
E. faecium	E. faec T1 & T2	E. faec T1 & T3	E. faec T1 & T2	-	-	E. faec T1 & T3	E. faec T1 & T2
E. coli	E. coli T1 & T2	-	E. coli T1 & T2	-	-	E. coli T1 & T2	E. coli T1 & T2

**HSV I**	HSV I T1 & T2	-	HSV I T1 & T2	HSV_01<br>HSV_02<br>HSV_03<br>HSV_04<br>HSV I T1<br>HSV_06<br>HSV_07<br>HSV_08<br>HSV I T2<br>HSV_10	HSV_01<br>HSV_02<br>HSV I T1<br>HSV_06<br>HSV_07<br>HSV_08<br>HSV I T2<br>HSV_10	-	HSV I T1 & T2
K. pneumoniae	K. pneum. T1 & T2	K. pneum. T1 & T2	K. pneum. T1 & T2	-	-	K. pneum. T1 & T2	K. pneum. T1 & T2
P. aeroginosa	P. aerug T1 & T2	P. aerug T1 & T2	P. aerug T1 & T2	-	-	-	P. aerug T1 & T2
S. aureus	S. aureus T1 & T2	S. aureus T1 & T2	S. aureus T1 & T2	-	-	-	S. aureus T1 & T2
**16S**	16S	16S	16S	-	-	-	16S
**AMR**	-	-	cat1_V2<br>ermB<br>mecA_V2<br>mecC<br>qnrA1<br>strA-APH(3'')<br>tetA<br>tetB<br>vanA<br>vanB<br>AAC(6)-Ib<br>TEM116<br>ndm1<br>OXA48<br>blaCTX-M-15_V2<br>blaKPC-2	-	-	cat1_V1<br>ermB<br>mecA_V1<br>mecC<br>qnrA1<br>strA-APH(3'')<br>tetA<br>tetB<br>vanA<br>vanB<br>AAC(6)-Ib<br>TEM116<br>OXA48<br>blaCTX-M-15_V1<br>blaKPC-2	cat1_V2<br>ermB<br>mecA_V2<br>mecC<br>qnrA1<br>tetA<br>tetB<br>vanA<br>vanB<br>AAC(6)-Ib<br>TEM116<br>ndm1<br>OXA48<br>blaCTX-M-15_V2<br>blaKPC-2

Supplement table S2. Raw sequencing and mapping data for technical validation. Mapping on full target genome.

Spike-In	Pathogen		Human	
	Mapped reads	portion all reads [%]	Mapped reads	portion all reads [%]
S. aureus	144,613	7.05375591	1,786,390	87.1343449
P. aeruginosa	830,584	25.4745282	2,297,695	70.4717356
K. pneumoniae	613,937	37.2932865	1,031,292	62.6453008
**HSV I**	18,424	16.3754333	91,532	81.3545463
E. coli	23,888	32.4283232	48,803	66.2508145
E. faecium	120,240	60.1795778	78,440	39.2588663
C. albicans	1,550	0.07287955	1,989,341	93.5369478
B. fragilis	235,297	16.6421593	1,099,192	55.2896548

Supplement table S3. Characteristics of used clinical samples. Timepoint reflects days after ICU admission. BC, blood culture.

Sample	Septic status	Timepoint [days]	Age [years]	Sex	Localization	BC result	Clinical Metagenomics [21]
**FL_01**	sepsis	14	57	male	Abdominal (peritonitis)	Negative (VRE OP smear)	B. fragilis, E. faecium, E. coli, H. influenzae, S. epi-dermidis
**FL_02**	sepsis	2	74	male	Abdominal (peritonitis)	Negative (VRE ab-dominal smear)	E. faecium
**FL_03**	sepsis	0	41	male	Abdominal (peritonitis)	E. coli	E. coli
**FL_04**	sepsis	1	75	male	Abdominal (peritonitis)	negative	B. thetaiotaomicron, B. ani-malis, E. faecium, E. coli, K. pneumonaie, L. fermentum, V. parvula
**FC_01**	sepsis	7	78	female	Abdominal (peritonitis)	negative	E. faecium, Human herpes-virus 1
**FC_02**	sepsis	28	66	male	Abdominal	negative	K. pneumoniae, P. mendo-cina, S. aureus
**FC_03**	OP control	1	NA	NA	-	-	-
**FC_04**	Healthy	-	NA	NA	-	-	-
**FC_05**	OP control	2	NA	NA	-	-	-
**FC_06**	sepsis	28	57	male	Abdominal (peritonitis)	Negative (VRE wound swab)	B. fragilis, B. thetaiotaomi-cron, E. faecium, H. influ-enza, Human herpesvirus 1

Supplement table S4. Raw sequencing and mapping data for selected clinical samples on MinION Flow Cell. Reads considered with mapq >4, all human reads accumulated, pathogen reads per target amplicons accumulated. FC, Flow Cell.

FC samples	FC_01	FC_02	FC_03	FC_04	FC_05	FC_06
H. sapiens	127,496	9,795	150,899	7,877	68,041	113,028
B. fragilis	0	0	0	0	0	2,262
C. albicans	0	0	0	0	0	0
CARD	0	0	0	0	0	832
E. coli	0	0	0	0	0	662
E. faecium	18	0	0	0	0	1
**HSV I**	101	0	51	0	0	520
K. pneumoniae	0	0	0	0	0	823
P. aeruginosa	0	0	0	0	0	0
S. aureus	0	0	0	0	0	0

Supplement table S5. Raw sequencing and mapping data for selected clinical samples on MinION Flongle. Reads considered with mapq >4, all human reads accumulated, pathogen reads per target amplicons accumulated. Igκ reads resulted from mapping on full target genome. FL, Flongle.

FL samples	FL_01	FL_02	FL_03	FL_04	FL_04-single
Igκ	6,474	760	8,104	9,64	8,692
H. sapiens	42,572	23,975	98,992	113,712	54,278
B. fragilis	1,603	1	1	1	0

C. albicans	0	0	0	0	0
**CARD**	1,562	12	1,187	2	54
E. coli	1	0	708	114	219
E. faecium	1,048	51	0	42	90
K. pneumoniae	0	0	0	0	0

Supplement table S6. Approximate duration of SUPSETS workflow. h, hours; min, minutes.

Step	Protocol
cfDNA isolation	2 h
End-prep and adapter ligation	3 h
Template generation	50 min
Purification	20 min
Amplification	1 h 50 min
Library preparation	3 - 4 h
Sequencing	5 min - 24 h
Total	11 - 12 h<br>5 min - 24 h sequencing

Supplement table S7. Approximate costs and price per sample of SUPSETS. For multiplexing on a MinION Flow Cell, 10 samples per run are calculated. For MinION Flongle experiments, costs for one single sample per analysis are depicted. State of prices: 12/2023.

Reagent	Selling price	Price per sample FC (10 samples)	Price per sample FL (1 sample)
**cfDNA isolation kit e.g. EZ1&2 ccfDNA Kit (48)**	1000 € / 48 reactions	20.83 €	20.83 €
**AmpliTaq Gold 360 Polymerase**	1,304 € / 1,000 U	5.22 €	5.22 €
**Primer / Adapter**	-	~ 0.1 €	~ 0.1 €
**AMPure XP Beads**	1,200 € / 60 mL	~ 2 - 3 €	~ 2 - 3 €
**Ligation Sequencing Kit V14 SQK-LSK114**	570 € / 6 reactions	-	95 €
**Native Barcoding Kit 24 V14 SQK-NBD114.24**	665 € / 6 reactions	11 € (for 6 x 10 samples)	-
**MinION Flongle R.10.4.1**	797 € / 12 Flongles	-	66.42€
**MinION Flow cell R.10.4.1**	855 €	85 € (for 10 samples)	-
**NEB Blunt/TA Ligase Master Mix**	438 € / 250 reactions	1.75 €	1.75 €
**dNTP Mix (10 mM)**	100 € / mL	0.2 €	0.2 €
**NEB Quick Blunting Kit**	378 € / 100 reactions	3.78 €	3.78 €
**NEBNext FFPE DNA Repair Mix**	642 € / 96 reactions	6.69 €	6.69 €
**NEBNext Ultra II End Repair/dA-Tailing Module**	886 € / 96 reactions	9.23 €	9.23 €
**NEBNext Quick Ligation Reaction Buffer (5x)**	47 € / 2 mL	0.24 €	0.24 €
**T4 Quick Ligase (400 U/µL)**	288 € / 100,000 U	10 - 12 €	10 - 12 €
**Total**		~ 160 €	~ 225 €
**Upfront costs:**			

**MinION Starter pack** (1 Mk1B, 1 Flow cell, 1 sequencing kit, 1 washing kit)	950 €		
**Optional:** cfDNA isolation device e.g. EZ2 Connect	30,000 €		

Supplement table S8. Reference genoms and corresponding TaxIDs.

Reference	Corresponding ID
H. sapiens	GRCh38
B. fragilis	NZ_CP069563.1
C. albicans	C_albicans_SC5314
E. faecium	NZ_CP039729.1
E. coli	NC_000913
HSV I	NC_001806.2
K. pneumoniae	NC_016845.1
P. aeruginosa	NC_002516.2
S. aureus	NC_007795.1

Supplement table S9. List of primers and target sequence.

Primer name	Target Primer sequence (5′ – 3′)	Adressed Gen	Adressed genomic region
**P. aerug T1**	GATCGAGTCGTTCATCGG	PA2338	NC_002516.2:2,581,960-2,582,147
**P. aerug T2**	CAGGATATCGCTCAACCG	PA1813	NC_002516.2:1,971,919-1,972,054
**S. aureus T1**	GTAGGTACATCATCGTTAAGGC	SAOUHSC_00060	NC_007795.1:64,011-64,883
**S. aureus T2**	GCGATTATTGTTGCTGAAGG	SAOUHSC_02279	NC_007795.1:2,110,362-2,110,725
**C.alb T1**	GGATTGCCTTGTCTAACCC	FGR14	Ca21chrR_C_albicans_SC5314:1,129,506-1,131,023
**C.alb T2**	GGTATTTGGGTTGCTGGG	-	Ca21chr1_C_albicans_SC5314:173,093-173,226
**C.alb T3**	CTGCCTCCATACTTTGTAGGC	SFI 1 2.0	Ca21chr7_C_albicans_SC5314:645,263-645,549
**K.pneu T1**	GTGGTGGAAGTCTACACCG	KPHS_05240	NC_016845.1:562,800-563,175
**K.pneu T2**	GGCTATGGGATGATATTGCC	KPHS_18590	NC_016845.1:1,929,825-1,930,019
**B.frag T1**	TCGGAAGGAGAGAACATCC	I6J55_RS14525	NZ_CP069563.1:3,565,788-3,566,174
**B.frag T2**	TCTATAGATGGCAACACACGG	I6J55_RS00215	NZ_CP069563.1:44,925-45,117
**E.faec T1**	AAGACGCACTAGCGAAAGC	sufD	NZ_CP039729.1:568,370-568,596
**E.faec T2**	GCAATTGTAGAAGGCGTGGC	pslx	NZ_CP039729.1:101,372-101,564
**E.faec T3**	GTCAATCTGGTCTGCCTCA	RecG	NZ_CP039729.1:101,067-101,280
**E.coli T1**	CAGAACCGCGCTAATT	cynR	NC_000913:358,005-358,231
**E.coli T2**	CTCGCGCGATTATCC	yejB	NC_000913:2,275,142-2,275,233
**HSV I T1**	GGTCGAAAGCGTACTTGGC	UL12	NC_001806.2:25,762-25,934
**HSV I T2**	GCAACTCGTAGCAAATAGGC	UL16	NC_001806.2:30,224-30,342
**HSV_01**	CTGTTGGGTTAGAATTTCGG	UL41	NC_001806.2:91,653-91,672
**HSV_02**	AACGTCTCCATCCATGCC	US8	NC_001806.2:141,973-141,990
**HSV_03**	AGTGATGTAAATGGTGTCCG	UL4/5	NC_001806.2:12,022-12,041
**HSV_04**	GTGGTTCATGTGTGTGCC	-	NC_001806.2:94,673-94,690
**HSV_06**	AAAGCAGTAACCAGGTCCG	-	NC_001806.2:91,065-91,083
**HSV_07**	AAACCTAAGGACAGCGGC	UL15/17	NC_001806.2:31,426-31,443

**HSV_08**	CGATGTTAATGGTGAACGC	UL15/17	NC_001806.2:31,479-31,497
**HSV_10**	ACCACCAGGTGTTTAACCC	UL44	NC_001806.2:97,225-97,243
**16S**	CCATGAAGTCGGAATCGC	16S	-
**Igκ**	AGAGCAGGACAGCAAGGAC	Igκ constant chain	NC_000002.12:88,857,361-88,857,683
**cat1_V1**	AAGCACAAGTTTTATCCGGC	cat1	gb|KC243783.1|+|1-2007|mecA
**cat1_V2**	AAGATGTGGCGTGTTACGG	cat1	gb|KC243783.1|+|1-2007|mecA
**blaCTX-M-15_V1**	TTGTTAGGAAGTGTGCCGC	blaCTX-M-15	gb|AY044436.1|+|1436-2311|CTX-M-15
**blaCTX-M-15_V2**	GGAGACGAAACGTTCCG	blaCTX-M-15	gb|AY044436.1|+|1436-2311|CTX-M-15
**ndm1**	ACATCGCTTTTGGTGGC	ndm1	gb|FN396876.1|-|2407-3219|NDM-1
**TEM116**	TGAGGCACCTATCTCAGCG	TEM116	gb|U36911.1|+|1430-2290|TEM-116
**mecA_V1**	ACTACGGTAACATTGATCGC	mecA	gb|KC243783.1|+|1-2007|mecA
**mecA_V2**	AATCGATGGTAAAGGTTGGC	mecA	gb|KC243783.1|+|1-2007|mecA
**mecC**	CATCACCAGGTTCAACCC	mecC	gb|FR821779.1|-|35681-37678|mecC
**strA-APH(3'')**	GCATTCTGACTGGTTGCC	strA-APH(3'')	gb|AF313472.2|+|15594-16397|APH(3'')-Ib
**ermB [46]**	GAACATCTGTGGTATGGCG	ermB	gb|AF242872.1|+|2132-2878|ErmB
**OXA48**	GCACGTATGAGCAAGATGC	OXA48	gb|AY236073.2|+|2188-2985|OXA-48
**blaKPC-2**	TCTGACAACAGGCATGACG	blaKPC-2	gb|AY034847.1|+|6-887|KPC-2
**vanA**	CCAATACCGCACAACCGA	vanA	gb|M97297.1|+|6980-8010|vanA
**vanB**	GAAGTGGATCAAATCCGGC	vanB	gb|KF823969.1|+|5111-6139|vanB
**qnr1**	CAATGCCAATCTCAGCGG	qnr1	gb|DQ831141.1|+|8922-9578|QnrA1
**tetA**	AGGCAAGCAGGATGTAGC	tetA	gb|AF534183.1|+|2971-4245|tet(A)
**tetB**	CAAAGGCTTGGAATACTGAGTG	tetB	gb|AB089595.1|+|1-1206|tet(B)
**AAC(6)-Ib**	TCATATCGTCGAGTGGTGG	AAC(6)-Ib	gb|JQ808129.1|+|634-1188|AAC(6')-Ib
